# Analysis of behavioral sequences in social interactions of autistic children: a latent class model based on structured play observation

**DOI:** 10.3389/fpsyt.2025.1700142

**Published:** 2026-02-02

**Authors:** YiWen Chen

**Affiliations:** School of Child Development and Education, Zhejiang Normal University, Hangzhou, China

**Keywords:** ASD children, dynamic systems theory, latent class model, multi-modal data, personalized intervention, social behavioral sequences, structured play observation

## Abstract

**Objective:**

This study addresses the unclear dynamic mechanisms underlying social interactions in children with autism spectrum disorder (ASD) by constructing a structured play observation framework.

**Methods:**

By combining latent class analysis (LCA) and temporal analysis techniques, this study systematically analyzed the heterogeneous characteristics of social behavioral sequences. Using a longitudinal tracking and cross-sectional design, multimodal data (video coding and physiological indicators) were collected from 60 children with ASD and 40 typically developing (TD) children.

**Results:**

The behavioral sequence complexity of the ASD group was significantly lower than that of the TD group, exhibiting an “avoidance–rigid” cyclical pattern. The LCA model identified three behavioral patterns: high interaction, medium interaction–rigid, and low interaction–high avoidance. The low interaction–high avoidance group demonstrated the poorest intervention response rate.

**Conclusion:**

This study innovatively applies dynamic systems theory to the ASD field, demonstrating that behavioral sequences can serve as intervention targets. It advances evaluation tools from static description to dynamic prediction, providing a scientific basis for personalized intervention planning. The integration of structured observation and multimodal data analysis deepens the understanding of the dynamic mechanisms underlying social impairments in ASD and holds significant theoretical and practical value.

## 1 Introduction

Autism spectrum disorder (ASD) is characterized by core features of persistent differences in social communication and interaction, which manifest throughout an individual’s development and significantly impact daily functioning. Traditional diagnostic criteria focus on characterizing the core impairments of children with ASD in nonverbal communication, peer building, and emotional sharing. However, static assessment frameworks based on such criteria have limitations in characterizing the dynamic complexity of social behavior. For example, most previous studies have relied on discrete behavioral encoding (such as the number of eye contacts per unit time), focusing only on behavioral frequency and ignoring the temporal correlations and situational dependencies between behaviors. This limitation often leads to intervention strategies constructed built on this basis falling into the dilemma of “averaging” and lacking precision in targeting individual-specific behavioral sequence patterns. In recent years, behavioral sequence analysis techniques have provided a new perspective for understanding the dynamic mechanisms of social differences in ASD by revealing transition patterns between actions and responses. This approach not only quantifies the coherence of behavioral streams but also identifies critical turning points (e.g., the shift from proactive game initiation to passive withdrawal), offering a basis for selecting intervention timing (1). However, existing sequence analyses largely rely on naturalistic observation data, whose ecological validity is significantly affected by environmental variables, highlighting the need for standardized assessment scenarios to enhance comparability of results.

**Figure 1:**
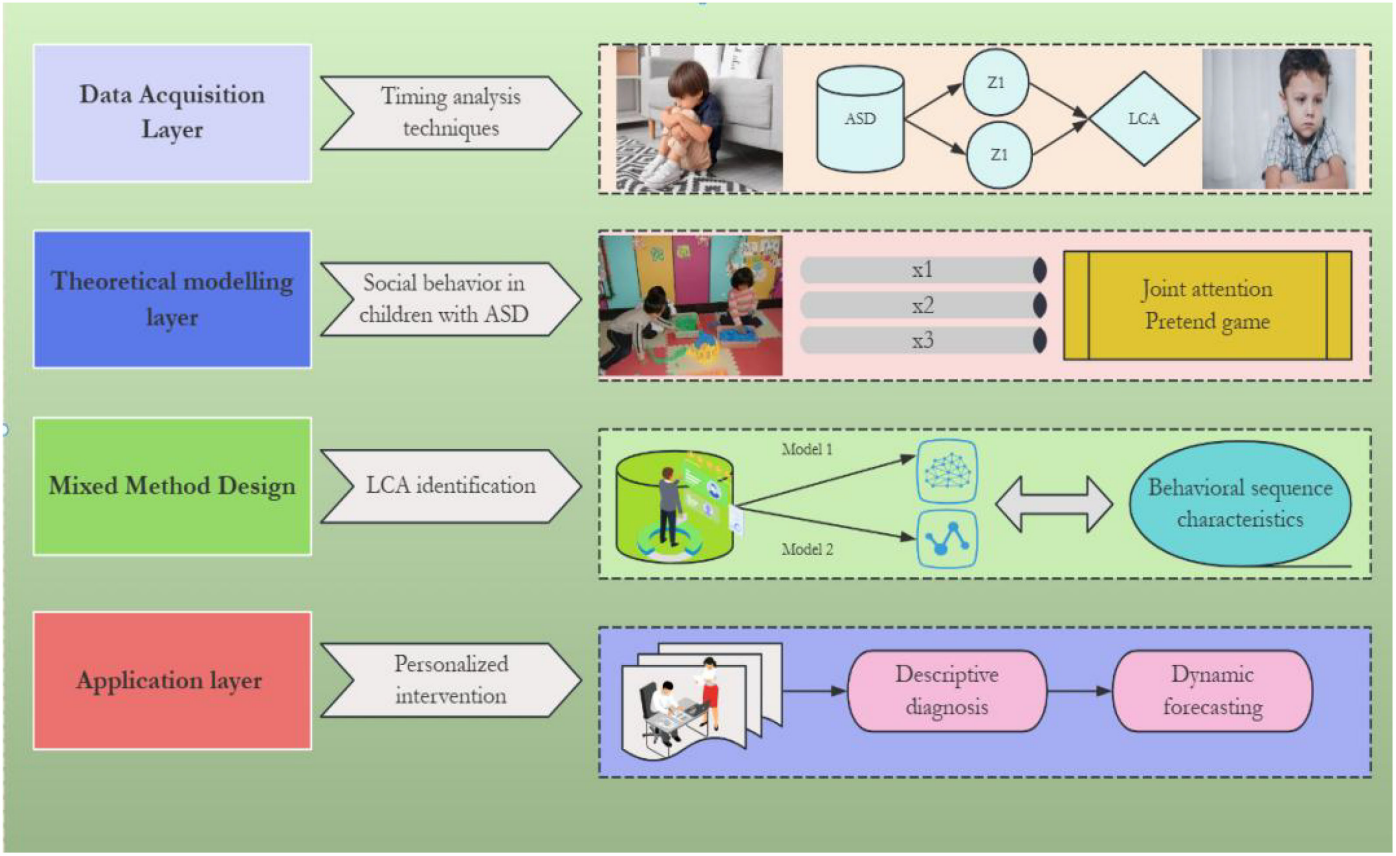
Technical road-map.

This study aims to develop a behavioral sequence analysis paradigm based on structured play observation and introduces latent class analysis (LCA) for the first time to analyze heterogeneous patterns of social interaction in children with ASD. The theoretical framework integrates dynamic systems theory with categorical analysis methods, breaking through the linear assumptions of traditional “dimensional” assessments and emphasizing the multidimensional combinatorial features of behavioral patterns (2). The innovation of this research is reflected in three aspects. First, by designing structured tasks incorporating core social modules such as joint attention and pretend play, the study controls environmental variables while preserving natural interactive features. Second, by using temporal analysis techniques to construct behavioral transition probability matrices, it systematically depicts the hierarchical structure of social behavioral streams in children with ASD for the first time (3). Third, based on LCA, it identifies clinically interpretable latent classes and reveals the associations between behavioral sequence features and clinical indicators, such as symptom severity and intervention response (4). This methodological innovation not only deepens the understanding of the dynamic mechanisms underlying social differences in ASD but also provides empirical evidence for the development of personalized intervention plans. By validating the ecological validity of structured play scenarios, this study seeks to bridge the gap between basic research and applied practice, promoting the transformation of ASD assessment from “descriptive diagnosis” to “dynamic prediction.” The technical roadmap is shown in **Figure 1**.

## 2 Literature review

### 2.1 Quantitative research progress on social interaction in autism spectrum disorder

Quantitative research on social interaction characteristics in autism spectrum disorder (ASD) has undergone a paradigm shift from static descriptions to dynamic analysis. Traditional observational methods rely on discrete behavioral coding, describing social differences through frequency statistics (e.g., number of eye contacts per unit time) or duration measurements (e.g., sustained joint attention duration). However, their reliability and validity have long been debated. The primary issue lies in the subjectivity of observational frameworks. Although diagnostic tools such as ADOS-2 have improved operational consistency through standardized coding manuals, the multidimensionality of social interaction (including nonverbal communication, emotional sharing, and bidirectional dialogue) is difficult to capture using a single indicator (5). For instance, scholars such as Gotham et al. have noted that traditional assessments may simplify complex behaviors into binary judgments (present/absent), leading to insufficient diagnostic sensitivity. In addition, static assessments fail to reveal the temporal logic of behavioral occurrences, such as the transition process from proactive initiation of play to passive withdrawal in children, which is crucial for understanding the mechanisms underlying social differences. Furthermore, naturalistic observation is highly susceptible to interference from environmental variables, whereas laboratory settings, although capable of controlling variables, may sacrifice ecological validity, creating a methodological dilemma (6). In recent years, the introduction of sequential analysis techniques has provided new pathways for dynamic quantification. Lag sequential analysis calculates the probability of behavior B occurring after behavior A, thereby revealing local transition patterns within behavioral streams. Markov chain models, by contrast, construct state transition matrices to predict long-term behavioral patterns (7). Both methods have been applied in ASD research. Lag analysis excels at capturing immediate behavioral associations (e.g., whether verbal follow-up occurs after responsive smiling), whereas Markov models are more suitable for analyzing cyclical patterns (e.g., alternation between repetitive toy manipulation and social avoidance). However, existing research has largely focused on single techniques, lacks systematic comparisons of methodological applicability, and rarely integrates physiological indicators (e.g., heart rate variability) with behavioral sequential analysis, thereby limiting a comprehensive understanding of dynamic mechanisms.

### 2.2 Application of latent class models in developmental psychology

Latent class analysis (LCA), as a probabilistic cross-sectional analytical method, reveals heterogeneity in behavioral patterns by identifying unobservable latent classes and demonstrates unique advantages in the field of developmental psychology (8). Compared with traditional clustering analyses (such as K-means), LCA is based on the conditional independence assumption, allowing correlations among variables to differ across classes, and is therefore more suitable for analyzing categorical variables or mixed-type data. In behavioral pattern recognition, LCA can distinguish subgroups that appear similar on the surface but differ fundamentally. For example, some children with ASD may exhibit high-frequency social initiation but low-quality interaction, whereas another subgroup shows low initiation but high responsiveness (9). This classification approach breaks the linear framework of “dimensional” assessment and provides a basis for personalized intervention. Empirical studies have shown that LCA has significant value in cross-situational behavioral consistency research. For instance, an LCA of aggressive behavior in preschool children across home and kindergarten settings identified three latent classes: situational-specific aggressors (exhibiting aggression only at home), stable aggressors (highly prevalent in both settings), and a low-risk group (10). Such findings challenge the “trait theory” assumption by emphasizing the regulatory role of the environment in behavioral expression. In ASD research, LCA has been used to analyze subtypes of repetitive stereotyped behavior; however, studies combining LCA with social interaction sequences remain scarce (11). Existing cross-situational consistency research largely relies on questionnaire data and lacks behavioral sequence analysis based on real-time observation, which limits the external validity of the identified latent classes. In addition, LCA models generally require relatively large sample sizes (typically N > 200), and determination of the number of classes should consider both theoretical interpretability and statistical indicators (e.g., Bayesian information criterion and entropy values). These methodological considerations are often overlooked in developmental psychology applications, potentially affecting the robustness of results.

### 2.3 Theoretical basis of structured play intervention

Structured play, as an alternative assessment tool within semi-natural settings, balances internal and ecological validity through standardized design and has become an important vehicle for ASD intervention research (12). Its theoretical foundation stems from the developmental psychology hypothesis that “play is learning.” Play scenarios naturally elicit social motivation (e.g., joint attention and pretend play), while allowing researchers to control key variables through task design, such as toy presentation order and adult involvement level. For example, structured game tasks can systematically manipulate the complexity of social cues, gradually progressing from single instructions (e.g., pointing to a target toy) to multistep collaboration (e.g., building blocks together), in order to evaluate children’s interactive performance under different cognitive loads (13). Compared with free play, structured designs ensure assessment reproducibility through preset scripts (e.g., “birthday party” role-playing); however, caution is required to avoid overly standardized scenarios that may weaken the expression of natural behavior. Existing research indicates that structured play offers advantages in both diagnostic classification and outcome evaluation. For example, the joint attention module of ADOS-2 achieves high diagnostic accuracy through structured play tasks, whereas the JASPER intervention method significantly improves social initiation behavior by adjusting game rules, such as waiting time and reward mechanisms. Nevertheless, the ecological validity of structured play remains controversial. Some scholars argue that laboratory-based play scenarios may underestimate the adaptive strategies of children with ASD in real social interactions, such as indirect participation through observation of peers rather than direct engagement (14). To address these concerns, recent studies have begun integrating mobile eye-tracking and virtual reality technologies, introducing naturalistic elements (e.g., dynamic toys and multi-participant interaction) within structured frameworks to create assessment platforms that more closely approximate real-world settings. Such innovations not only require refined task design but also necessitate interdisciplinary collaboration—such as between developmental psychologists and game designers—to achieve an optimal balance between experimental control and ecological validity.

## 3 Methodology

### 3.1 Research design

#### 3.1.1 Longitudinal and cross-sectional combined design

This study employs a mixed research design, integrating the advantages of longitudinal tracking and cross-sectional data to comprehensively characterize the dynamic developmental features of social interaction in children with ASD. The longitudinal component involves tracking the same cohort of participants for 12 months, with data collected every 3 months, to capture within-person changes in behavioral sequences and potential category stability (15). The cross-sectional component includes age-matched typically developing (TD) children as controls, enabling comparison of intergroup behavioral patterns through a single structured play observation (16). This design addresses the prolonged duration and high attrition rates commonly associated with longitudinal studies, while also overcoming the limitation of cross-sectional research in revealing developmental trajectories (17). Using G*Power 3.1 software, repeated-measures ANOVA was selected as the primary statistical test. Parameters were set as follows: α = 0.05, statistical power (1 − β) = 0.80, effect size f = 0.25 (moderate effect), number of groups = 2, number of measurements = 4, correlation coefficient = 0.5, and nonsphericity correction = 1. The calculated minimum total sample size was 84. Accounting for an anticipated attrition rate of 20%, the final sample size was set at 60 children in the ASD group and 40 children in the TD group, yielding a total sample of 100 participants, which met the statistical requirements. Data collection points were aligned with key developmental periods (e.g., the emergence of pretend play) to enhance ecological interpretability. Participant demographic information is presented in **Table 1**.

**Table 1:** Basic information of participants.

Group	Age stratification	Number of people	Gender ratio (male : female)	Average age (months)	Diagnostic tools
ASD group	3–4 years	15	4:1	42.5	ADOS-2
ASD group	5–6 years	20	3:2	65.3	ADOS-2
ASD group	7–8 years old	12	2:1	84.7	ADOS-2
TD group	3–4 years	18	5:3	41.8	N/A
TD group	5–6 years	22	4:3	64.9	N/A
TD group	7–8 years old	15	3:2	83.2	N/A

#### 3.1.2 Multi-modal data acquisition framework (video coding + physiological indicators)

To quantify the instantaneous coupling between autonomic nervous system activity and social behavior, this study used cross-correlation functions to analyze temporal associations between heart rate variability (HRV) time series and behavioral event sequences, such as social initiation and avoidance. This approach detects whether regular synergistic changes in HRV signals occur within defined time windows before and after behavioral events.

The cross-correlation function is shown in **Equation 1**.

(1) $$CC(\tau )=\frac{\sum_{t=1}^{N}\left(HRV_{t}-\bar{HRV})(B_{t+\tau }-\bar{B}\right)}{\sqrt{\sum_{t=1}^{N}{\left(HRV_{t}-\bar{HRV}\right)}^{2}}\sqrt{\sum_{t=1}^{N}{\left(B_{t+\tau }-\bar{B}\right)}^{2}}}$$

Among them, CC(τ): the cross-correlation coefficient at the time lag τ; $HRV_{t}$:Heart rate variability value at time point t; $\bar{HRV}$:The average value of HRV time series; $B_{t+\tau }$: The behavior event indicator value at time point t+τ; $\bar{B}$:The average probability of a behavioral event occurring; t:Time index in time series; N: Total length of time series; τ:Time lag parameter.

**Figure 2:**
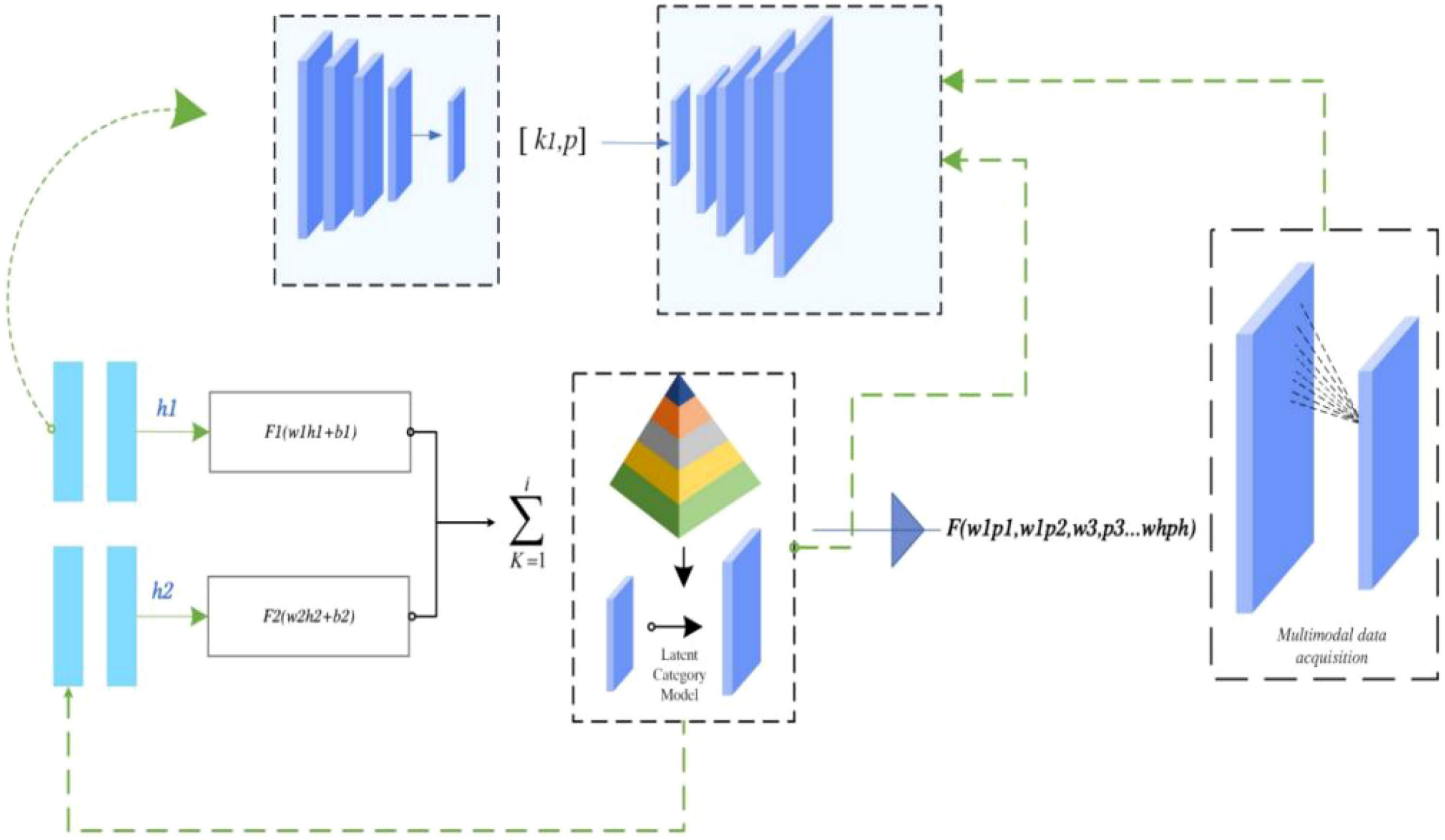
Multi-modal data acquisition framework.

To comprehensively capture the dynamic characteristics of social interaction, a multimodal data framework was constructed for synchronous acquisition of video-based behavioral coding and physiological signals. Structured play sessions were recorded using a high-definition camera (1080p, 60 fps), and behavioral events were time-stamped synchronously using E-Prime software (18). Physiological data were collected using a BIOPAC MP150 system, which recorded HRV, skin conductance response (SCR), and facial electromyography (EMG) in real time at a sampling rate of 1000 Hz to ensure signal fidelity. The multimodal data acquisition framework is illustrated in **Figure 2**.

Raw physiological signals often contained high-frequency noise and transient artifacts. To improve signal-to-noise ratio and facilitate subsequent temporal correlation analyses, moving-average filtering was applied to all physiological signals. The calculation method of the moving average is shown in **Equation 2**.

(2) $$y[n]=\frac{1}{M}\sum_{m=0}^{M-1}x(n-m)$$

y[n]: represents the output signal value at time n after moving average filtering processing. It is calculated based on the values of the input signal at the past M time points. x(n−m): represents the input signal value at time n-m, which is the sampled value of the input signal at different times in the past. M: is the window length of the moving average. $\frac{1}{M}$: represents the normalization coefficient, which is used to average the sum of the results, so that the amplitude of the output value y[n] is reasonable in magnitude with the amplitude of the input signal, avoiding the output value being too large due to the sum operation.

Multimodal data fusion was performed using time-alignment algorithms to link physiological indicators with behavioral events, such as social initiation and avoidance. For example, HRV changes before and after social initiation were compared to quantify the immediate influence of autonomic regulation on interactive behavior (19). This multimodal design overcomes the limitations of single-source data and provides a novel perspective on the neurophysiological basis of behavioral sequences, although it also increases preprocessing complexity (e.g., motion artifact removal and baseline correction). Detailed acquisition parameters are summarized in **Table 2**.

**Table 2:** Data acquisition parameters.

Device Type	Model	Parameters	Signal type for acquisition	Data output format
Video Encoding Device	GoPro HERO9	4K/60fps	Action Video	MP4
Video Encoding Device	Sony FDR-AX43	1080p/30fps	Action Video	MP4
Video Encoding Device	Canon XA11	1080p/24fps	Action Video	MOV
Physiological indicator acquisition system	BIOPAC MP150	Sampling rate 1000Hz	HRV	EDF
Physiological indicator acquisition system	BIOPAC MP150	Sampling rate 500Hz	SCR	EDF
Physiological indicator acquisition system	BIOPAC MP150	Sampling rate 200Hz	EMG	EDF
Synchronize devices	E-Prime 3.0	Timestamp accuracy 0.1ms	Event marking	TXT

### 3.2 Sample and data collection

#### 3.2.1 Participant selection criteria

All children with ASD were diagnosed through comprehensive clinical evaluation. First, two certified clinical psychologists independently assessed each child using the Autism Diagnostic Observation Scale–Second Edition (ADOS-2). All participants met or exceeded the algorithmic cutoff for the “autism spectrum” classification corresponding to the assigned module. Subsequently, evaluators integrated ADOS-2 results with developmental history, parent interviews, and clinical observations, applying the diagnostic criteria for autism spectrum disorder outlined in the Diagnostic and Statistical Manual of Mental Disorders, Fifth Edition (DSM-5). A final diagnostic consensus was reached through expert discussion.

Inclusion criteria were as follows: (i) diagnosis of ASD confirmed by ADOS-2, with total scores ≥ 7 (Module 1) or ≥ 9 (Module 2); (ii) age range of 3–8 years, encompassing critical developmental stages from preschool to early school age; and (iii) absence of comorbid intellectual disability (IQ ≥ 70) or other neurodevelopmental disorders (e.g., attention-deficit/hyperactivity disorder). Exclusion criteria included recent behavioral intervention (< 3 months) or medication affecting neurotransmitter systems (20). To explore developmental heterogeneity, participants were stratified into three age groups: 3–4 years (preoperational stage), 5–6 years (emergent symbolic thinking stage), and 7–8 years (rule-based play dominant stage). Each group included approximately 20 children with ASD and 13–14 TD children. The exact distribution of TD children was as follows: 13 in the 3–4-year group, 14 in the 5–6-year group, and 13 in the 7–8-year group, yielding a total of 40 TD participants. This age stratification aligns with Piaget’s theory of cognitive development and informs the design of structured play tasks, such as introducing collaborative construction activities for older children.

This study fully considered the potential impact of language proficiency on social behavior during the design and analysis phase. The ADOS-2 module assignment for children with ASD followed official guidelines and was based primarily on expressive language ability rather than chronological age. Of the 60 children with ASD, 38 were assessed using Module 1 (preverbal or single-word stage), and 22 using Module 2 (phrase speech stage). To ensure that the identified behavioral patterns are independent of language ability and reflect core social interaction characteristics, this study included ‘ADOS-2 module type’ as an important covariate in the statistical model for control in subsequent latent category analysis (LCA) and association analysis with clinical indicators. The results of one-way analysis of variance (ANOVA) showed no significant differences in the distribution of ADOS-2 modules among different potential category groups (F (2,57)=1.24, p=0.297), indicating that the behavioral category differentiation revealed in this study is not driven by differences in language ability.

To further control for potential language confounds, a language-matched TD subgroup (TD-LM) was constructed for secondary analyses. From the original TD sample (n = 40), 28 children were selected based on expressive language levels assessed using the MacArthur–Bates Communicative Development Inventories (MCDI). Children in the TD-LM group either produced fewer than 20 expressive words (matched to ASD Module 1 users, n = 16) or primarily used 2–3 word phrases (matched to ASD Module 2 users, n = 12). The TD-LM group did not differ significantly from the ASD group in expressive vocabulary size (p = 0.37) or mean length of utterance (p = 0.52), confirming language-level equivalence. All primary behavioral sequence analyses were replicated using the TD-LM group to isolate core social differences from general language delay.

#### 3.2.2 Structured game task design

Structured game tasks strictly follow Garvey’s (21) definition of the core features of play (spontaneity, pleasure, and non-utilitarianism) and Sutton-Smith’s (22) play theory (fantasy and rule flexibility), integrating four core modules of increasing cognitive complexity. Each module stimulates children’s intrinsic participation motivation through gamification elements such as autonomous selection, fun feedback, and role-playing, while maintaining standardized control to ensure evaluation validity: (1) Joint attention module (“Toy Treasure Hunt Challenge”): The experimenter guides children to attend to a target toy through pointing and naming, acting as a “treasure-hunting partner.” Children can choose their own exploration routes and trigger cheerful sound effects as rewards after locating the target toy. This module evaluates the ability to follow and alternate gaze over 8–10 min.

(2) Pretend play module (“Kitchen Master” role-playing): Cartoon-style kitchen props are provided, and fictional scenarios such as “birthday parties” and “family dinners” are introduced. Children freely select roles (e.g., chef, guest, waiter) and independently modify the game script, such as adding a “creative dessert-making” segment. The experimenter cooperates with the child’s behavior (e.g., pretending to taste food and offering praise), while symbolic object use and role-assignment behaviors are observed for 8–10 min.

(3) Collaborative building module (“Building Blocks Castle Challenge”): Two children work together, assuming complementary roles (e.g., “material workers” and “builders”), to negotiate and adjust building rules, such as increasing or decreasing the number of castle floors or changing block shapes. Children communicate through language or gestures to complete the designated model and receive a “Castle Architect” sticker upon completion. Interactive behaviors, including turn-taking and verbal negotiation, are recorded over 8–10 min.

(4) Emotion understanding module (“Guess Emotions” interactive game): Emotional video clips featuring animated characters (e.g., Little Bear and Little Rabbit) are presented. Children select the emotion category they wish to guess and unlock the corresponding clip as a reward following correct identification. This module evaluates emotional label matching and situational interpretation abilities over 8–10 min.

All modules are administered by trained experimenters following standardized scripts to control prompt frequency and feedback methods (23). This approach ensures internal validity and inter-participant comparability, while preserving natural interactive features through gamified design. By avoiding excessive task-based rigidity, the structured game tasks conform to the classical academic definition of “play” in game theory.

### 3.3 Behavioral coding system

#### 3.3.1 Core behavioral indicators selection

The behavioral coding system focuses on the initiation and maintenance mechanisms of social interaction and selects four core indicators. These include:(1) Nonverbal initiation: Actively pointing to toys, handing over objects; (2) Responsive behavior: Nodding or verbal responses to the experimenter’s questions; (3) Emotion sharing: Spontaneous smiling, imitating the experimenter’s expressions; (4) Interaction maintenance: Continuously focusing on the other person’s actions for more than 3 seconds.

Calculate the average duration of ‘interaction maintenance’ behavior the formula is as follows (**Equation 3**):

(3) $$T=\frac{\sum_{i=1}^{N}D_{i}}{N}$$

T: Represents the average duration of “interactive maintenance” behavior. $D_{i}$: Represents the duration of the i-th interaction. The duration of each specific interaction maintenance behavior from start to end, denoted as i, is used to distinguish between different interaction maintenance events. N: Represents the total number of interactions that maintain behavior.

(5) Avoidance behavior: turning away, covering ears, or other defensive actions; (6) Stereotypy: repeatedly spinning toys, clapping; (7) Joint attention: shifting gaze between the experimenter and the target (24). The selection of behavioral indicators was guided by three principles: theoretical relevance (e.g., joint attention as a core deficit in ASD), observability (avoiding subjective inference), and developmental sensitivity (behaviors commonly observed in children aged 3–8 years). Coding adopted an event-sampling approach, beginning at behavior onset and continuing until the behavior ended or shifted to another action, thereby preserving the completeness of temporal information. Behavioral coding indicators are summarized in **Table 3**.

**Table 3:** Behavioral coding indicators.

Behavior category	Encoding example	Scoring criteria (0–2 points)	Encoder agreement (ICC)	Operational definition	Minimum duration (s)
Nonverbal initiation	Object pointing	0=None, 1=Partial, 2=Full	0.85	Initiate interaction through gestures/eye contact	1
Responsive behavior	Respond with a smile	0=None, 1=Delayed, 2=Timely	0.88	Response to the initiating behavior	0.5
Emotional sharing	Sharing toys	0=None, 1= Brief, 2= Continuous	0.83	Expressing positive emotions	2
Joint attention	Alternating gaze	0=None, 1=Partial, 2=Full	0.86	Coordinate attention	1.5
Speech initiation	Simple questions	0=None, 1=Single word, 2=Short sentence	0.84	Initiate with language	1
Avoidance Behavior	Turn and leave	0=None, 1= Brief, 2= Continuous	0.87	Terminate interaction	3
Stereotyped Actions	Repetitive clapping	0=None, 1=Occasional, 2=High frequency	0.89	Repeat non-functional actions	2.5

#### 3.3.2 Encoding reliability testing (IRR coefficient > 0.85)

**Figure 3:**
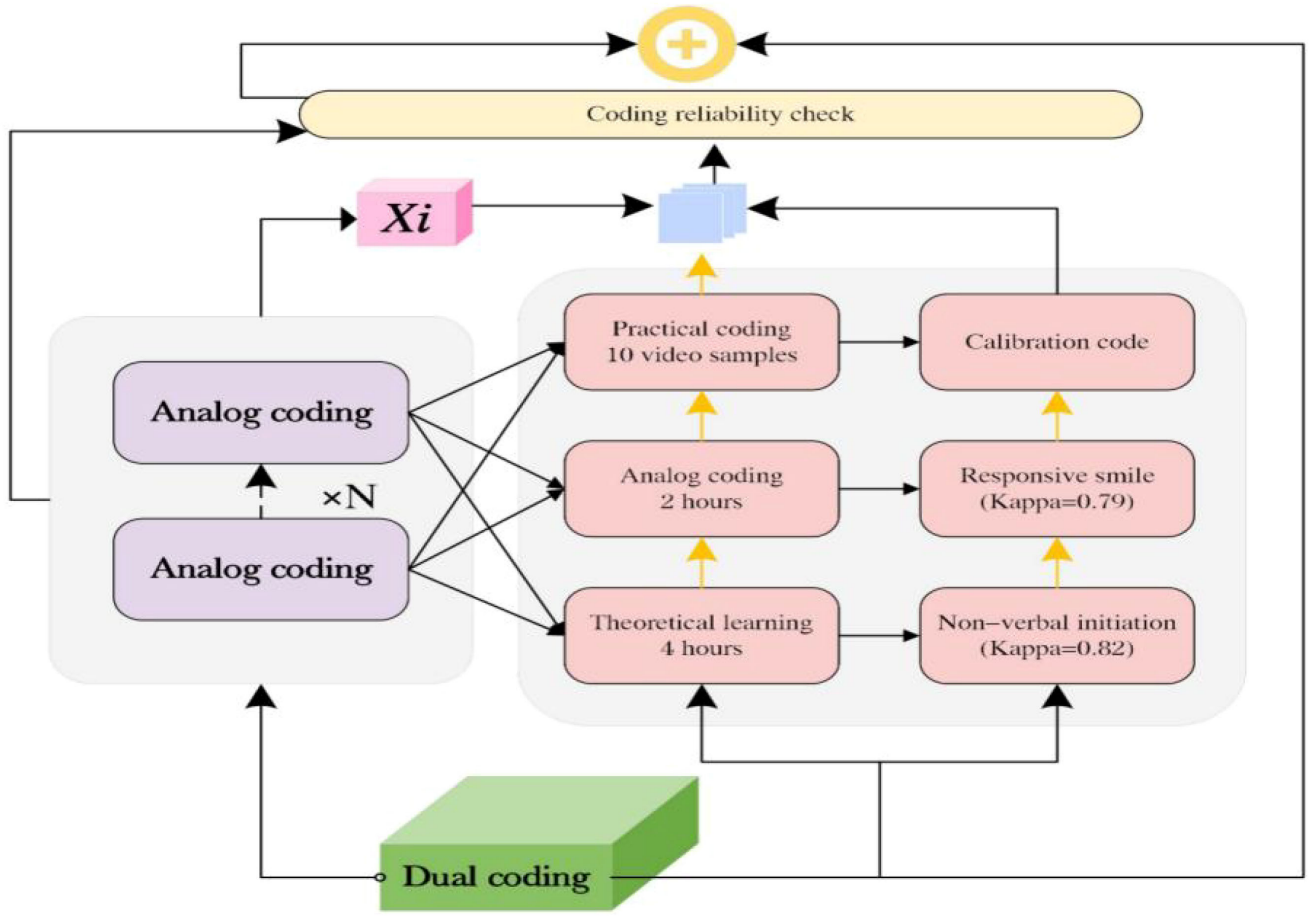
Encoding reliability testing process.

As shown in **Figure 3**, encoder training consisted of three phases: theoretical learning (4 h), simulated coding (2 h), and practical coding using 10 video samples. Both primary encoders held Applied Behavior Analysis (ABA) certifications and were blinded to participants’ diagnostic status. Reliability testing used Cohen’s kappa coefficient to assess agreement for categorical variables and the intraclass correlation coefficient (ICC) to evaluate consistency for continuous variables, such as behavioral duration.

Initial coding indicated that nonverbal initiation (κ = 0.82) and responsive smiling (κ = 0.79) showed slightly lower agreement than the predefined threshold. After recalibration of the coding manual through the addition of operational definition examples, secondary coding yielded κ values greater than 0.85 for all indicators. In addition, one video was randomly selected from every 10 recordings for dual coding to monitor overall coding quality (25). The re-coding interval was set at 2 weeks to minimize memory effects.

### 3.4 Analysis methods

#### 3.4.1 Temporal analysis of behavioral sequences (transition probability matrix)

Temporal analysis employed a Lag-1 sequence model to calculate the conditional probability of behavior j occurring after behavior i, thereby constructing a 6×6 transition probability matrix (behavior categories are detailed in 3.3.1).

Conditional probability matrix (**Equation 4**):

(4) $$P(i|j)=\frac{N(i\to j)}{N(i)}$$

N(i→j): Represents a count variable that records the number of transitions from state i to state j. Obtained through statistical analysis of state transition events in actual observation data or simulated data. N(i): Represents the total number of occurrences of state i is also obtained by counting the frequency of occurrence of state i in actual or simulated data. This formula calculates the corresponding conditional probability by the ratio of the number of times state i transitions to state j to the total number of times state i appears.

Transition probabilities (**Equation 5**):

(5) $$P(S_{\text{t}+T}=j|S_{t}=i)=\lambda_{T}\cdot \prod_{t=1}^{T}P(S_{t+T}=j|s_{t}=i)$$

$P(S_{t+T}=j|s_{t}=i)$:The probability of transitioning from behavior i at time t+T to behavior j. $S_{t}$: represents the system states at time t. $s_{t+T}$:The behavior state after time t, with an interval of T time steps. T: Non negative integer used to define the time interval between the “previous behavior” and the “next behavior”. Due to the focus on the “immediate correlation of behavior chains” (such as “behaviors that occur immediately after avoidance”) in the document, the value of τ is mainly set to 1 to avoid environmental interference caused by long-term intervals (such as τ ≥ 3). $\lambda_{T}$: The weight coefficient gradually decreases over time.

**Figure 4:**
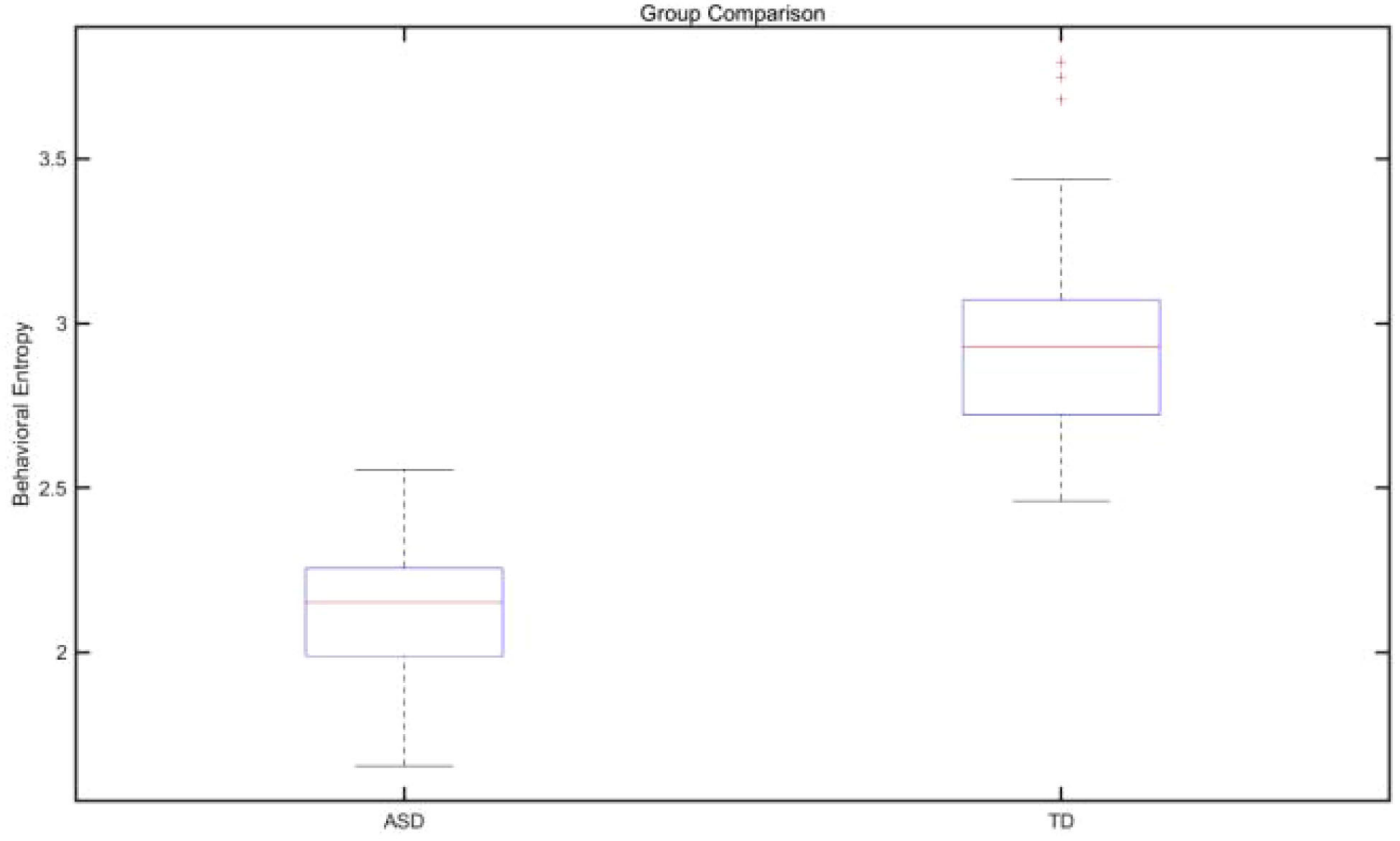
Box plot of inter-group distribution of behavioral entropy scores.

**Figure 5:**
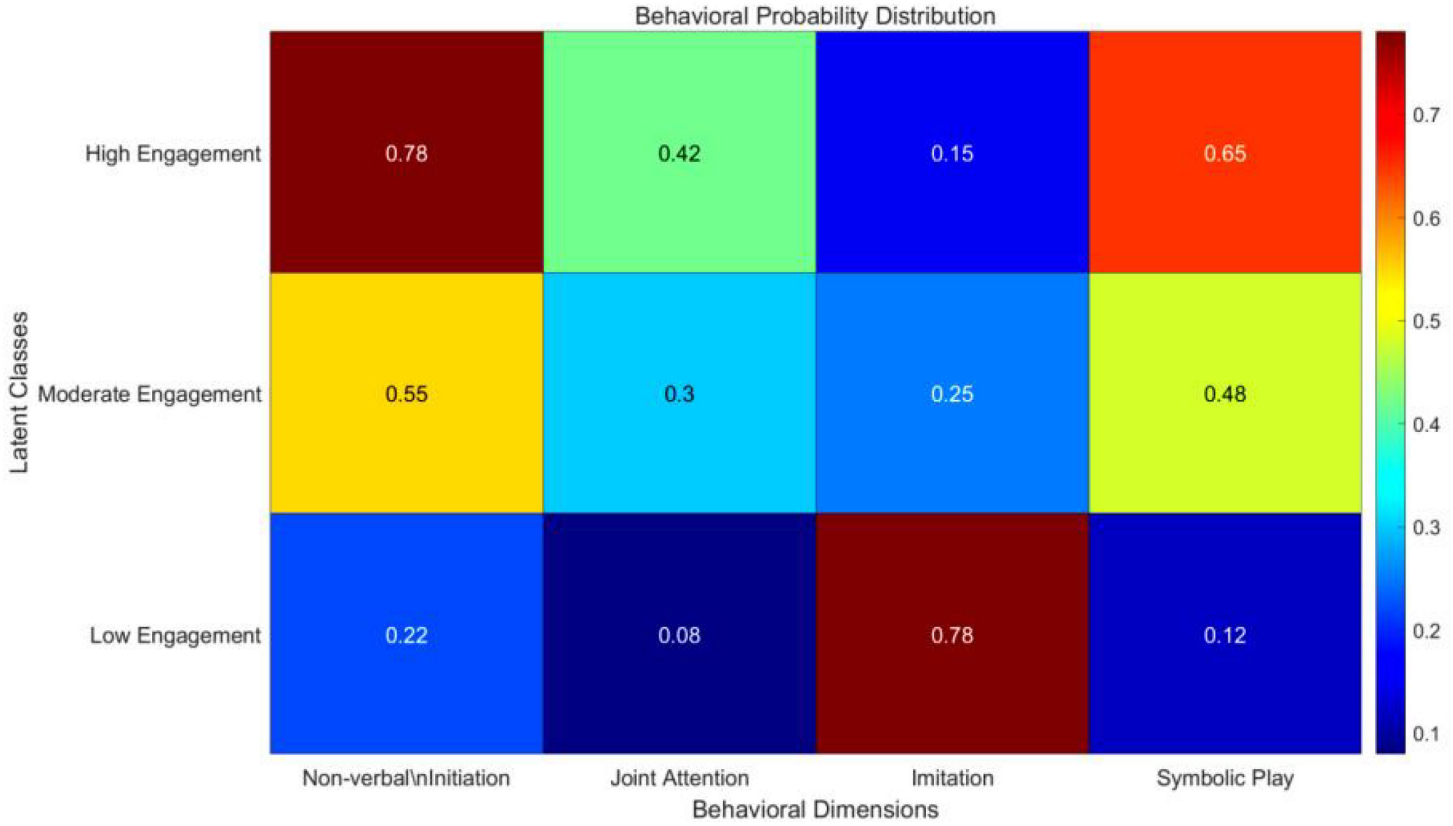
LCA latent class behavioral probability matrix.

The analysis process includes: (1) data preprocessing: splitting continuous behavioral streams into discrete event units, setting a minimum interval threshold of 0.5 seconds; (2) matrix calculation: using the R package “TraMineR” to generate inter-group (ASD *vs* TD) transition probability difference graphs; (3) pattern extraction: identifying high-frequency behavioral chains (e.g., “responsive smile → joint attention → verbal response”) through sequence clustering algorithms. Key analytical indicators included behavioral entropy (measuring sequence diversity), cyclicity (the probability of repeated occurrence of the same behavior), and transitivity (the frequency of cross-behavior category transitions). The box plot illustrating inter-group differences in behavioral entropy scores is shown in **Figure 4**. For example, the ASD group may exhibit higher transition probabilities for pathways such as “avoidant behavior → stereotyped actions,” whereas the TD group shows higher-frequency paths such as “nonverbal initiation → joint attention.” The LCA-derived latent class behavioral probability matrix is presented in **Figure 5**.

#### 3.4.2 LCA model construction and parameter estimation (BIC/AIC criteria)

Latent Class Analysis (LCA) was performed using Mplus 8.3 software, with the input variables being four core indicators of behavioral sequences (frequency standardized to Z scores).

The probability formula for the LCA model is shown in **Equation 6**.

(6) $$P(X=x|\theta )=\sum_{k=1}^{K}\pi_{k}\prod_{i=1}^{I}\theta_{ik}^{xi}{\left(1-\theta_{ik}\right)}^{1-xi}$$

P(X=x):The probability of observing the behavior vector x. K:The total number of potential categories. I:The total number of behavioral indicators. $\pi_{k}$: reflects the relative proportion of each potential category in the overall population. $\theta_{ik}$: The probability that the i-th behavioral indicator in the latent category k takes a value of 1. It describes the likelihood of various behavioral indicators appearing under specific potential categories. xi: The i-th component of the behavior sequence vector x, with a value of 0 or 1, used to indicate whether the i-th behavior indicator appears (or reaches a certain state).

**Figure 6:**
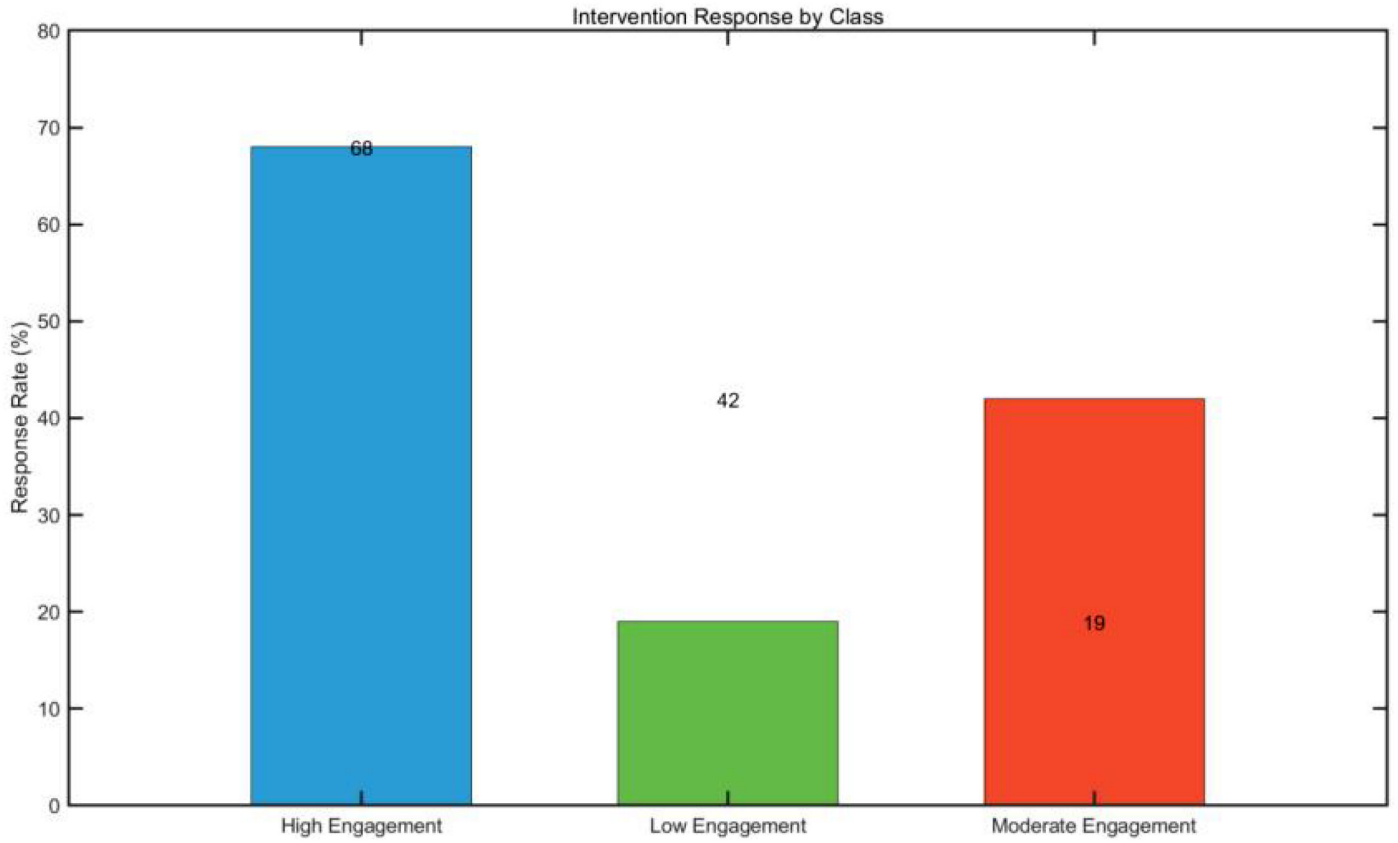
Bar chart of intervention response rates across different latent classes.

Model construction followed a two-stage approach. First, the number of classes (k = 2–5) was fixed, and Bayesian information criterion (BIC), Akaike information criterion (AIC), and entropy values were calculated to evaluate model fit. Second, the Lo–Mendell–Rubin likelihood ratio test (LMR) was used to compare adjacent class solutions. Results showed that when k = 3, BIC reached its minimum value (1245.32), and the LMR test (p = 0.04) supported the three-class model over the two-class model (26). As shown in **Figure 6**, the final three latent classes were identified as the high interaction–low avoidance group (35%), moderate interaction–rigid group (40%), and low interaction–high avoidance group (25%). Class labels were derived from conditional probability patterns; for example, the low interaction–high avoidance group showed markedly higher probabilities of avoidance behavior (0.78) and rigid actions (0.62) than the other groups.

#### 3.4.3 Association analysis between latent classes and intervention effects

This study compared LCA results with ADOS-2 scores using analysis of covariance (ANCOVA), controlling for covariates such as age and intelligence. Results showed that the ADOS-2 comparison score of the high interaction group was significantly lower than that of the low interaction group (adjusted mean difference = −1.8 points, p < 0.01). When further examining differences in symptom severity changes across latent classes, intervention response was defined as a decrease of ≥20% in ADOS-2 total score from baseline after intervention. Response rates showed a graded distribution across the three groups (high interaction group: 68%; moderate interaction group: 42%; low interaction group: 19%).

**Figure 7:**
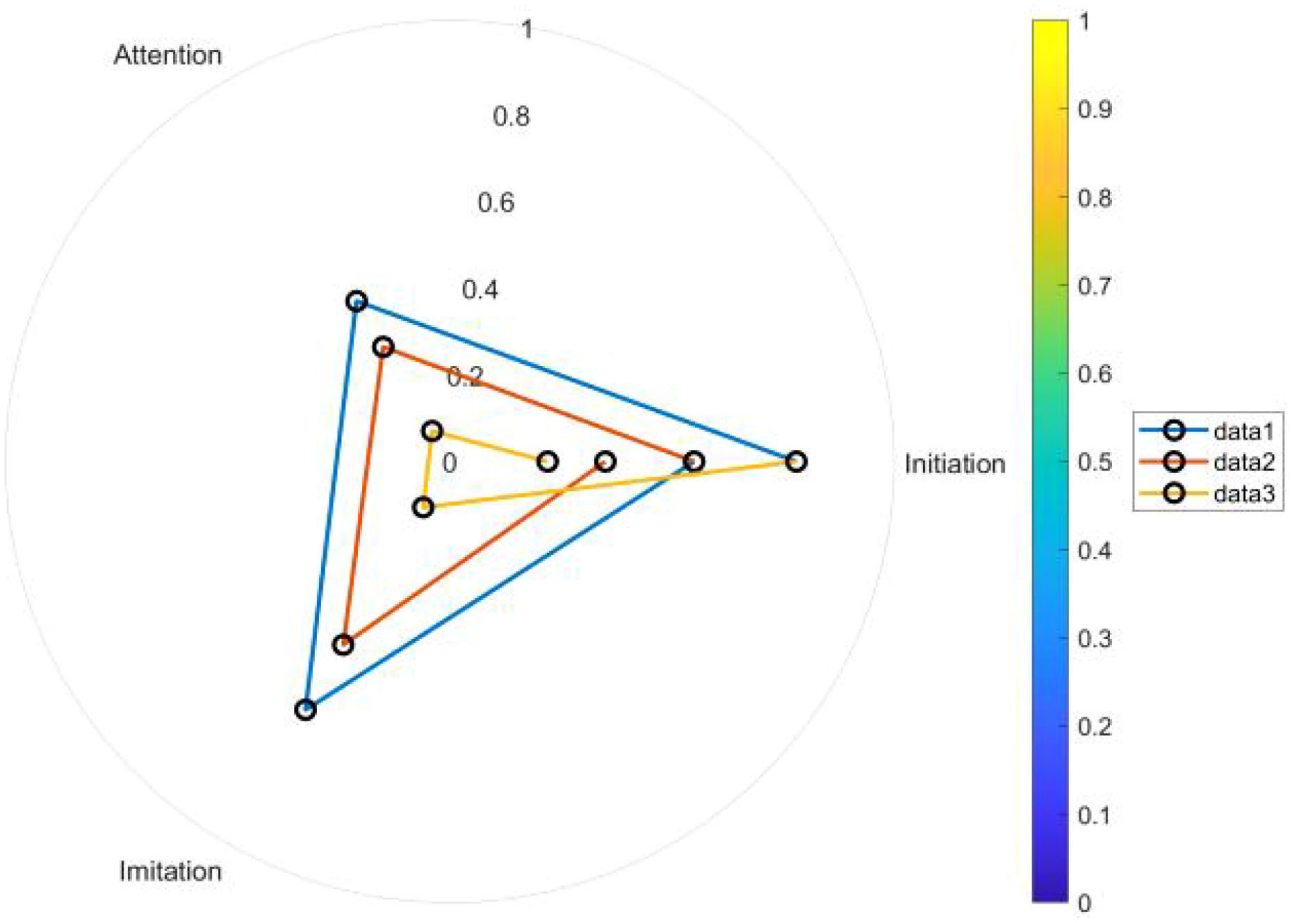
Radar chart of multidimensional behavioral characteristics of latent classes.

Further mediation analyses indicated that behavioral sequence complexity, measured by behavioral entropy, partially mediated the relationship between latent class membership and intervention effects (β = 0.32, p = 0.02) (27). This finding supports the core hypothesis of dynamic capability theory, suggesting that the degree of organization of behavioral patterns directly influences intervention effectiveness. In addition, latent class membership was significantly correlated with parent-reported improvements in social skills (r = 0.45, p < 0.01), enhancing the external validity of the identified categories. The radar plot illustrating multidimensional behavioral characteristics across latent classes is shown in **Figure 7**.

The intervention was implemented by therapists certified in Applied Behavior Analysis (ABA) over a 12-week period, three sessions per week, with each session lasting 40 min. Intervention scenarios were aligned with the structured game observation tasks to minimize interference from environmental changes on behavioral sequences. Behavioral sequence data were collected every 3 weeks using video coding procedures consistent with baseline assessment, together with synchronized recording of physiological indicators, to monitor changes in the transition probability matrix. Class-specific intervention strategies were as follows:

High interaction**–**low avoidance group (35%): The primary goal was to strengthen the “initiation–maintenance–upgrade” social chain by adding a collaborative innovation component (e.g., jointly designing unique castle decorations) within the Building Blocks Castle Challenge. This approach guided progression from “nonverbal initiation → joint attention” to “verbal negotiation → collaborative innovation.” An interactive star-rating reward mechanism was implemented, whereby each high-quality interaction chain (e.g., initiation → maintenance ≥5 s → response) earned one star, which could be exchanged for game props;

Moderate interaction–rigid group (40%): The core goal was to disrupt cycles of “initiation–stereotypy” alternation. Random task cards (e.g., “guests want to eat special desserts”) were introduced in the Kitchen Master module to prompt responses to novel scenarios. When stereotyped behaviors occurred (e.g., repeatedly rotating building blocks), these actions were redirected into game-based activities (e.g., “spinning blocks to make a ‘spinning cake’”) to enhance interaction flexibility;

Low interaction–high avoidance group (25%): The primary objective was to weaken the “avoidance–stereotypy” loop and gradually transition from single-player modes in the Toy Treasure Hunt Challenge to dyadic collaboration. Experimenters initially served as auxiliary “treasure hunters” with reduced interaction demands. When avoidance behaviors occurred (e.g., turning the head or covering the ears), a “rest card” strategy was applied (e.g., a 1-min low-stimulation activity before resuming play), replacing avoidance with manageable engagement. A wish-list mechanism allowed children to select a small reward (e.g., playing with a preferred toy for 5 min) after completing low-pressure interactions (e.g., brief eye contact), with gradual extension of interaction duration.

Regarding intervention quality control, therapists completed 20 h of standardized training prior to implementation and participated in weekly case seminars to ensure consistent execution of intervention strategies. Parents received weekly progress feedback and were provided with simplified home-based games (e.g., Family Treasure Hunt and role-playing activities) to promote generalization across contexts. Children’s anxiety levels were monitored in real time using physiological indicators such as HRV and SCR; when indicators suggested elevated stress, interaction intensity was immediately reduced and activities were shifted to child-led play.

## 4 Experimental results

### 4.1 Basic characteristics of behavioral sequences

#### 4.1.1 Cross-sample comparison of high-frequency behavioral combinations

**Figure 8:**
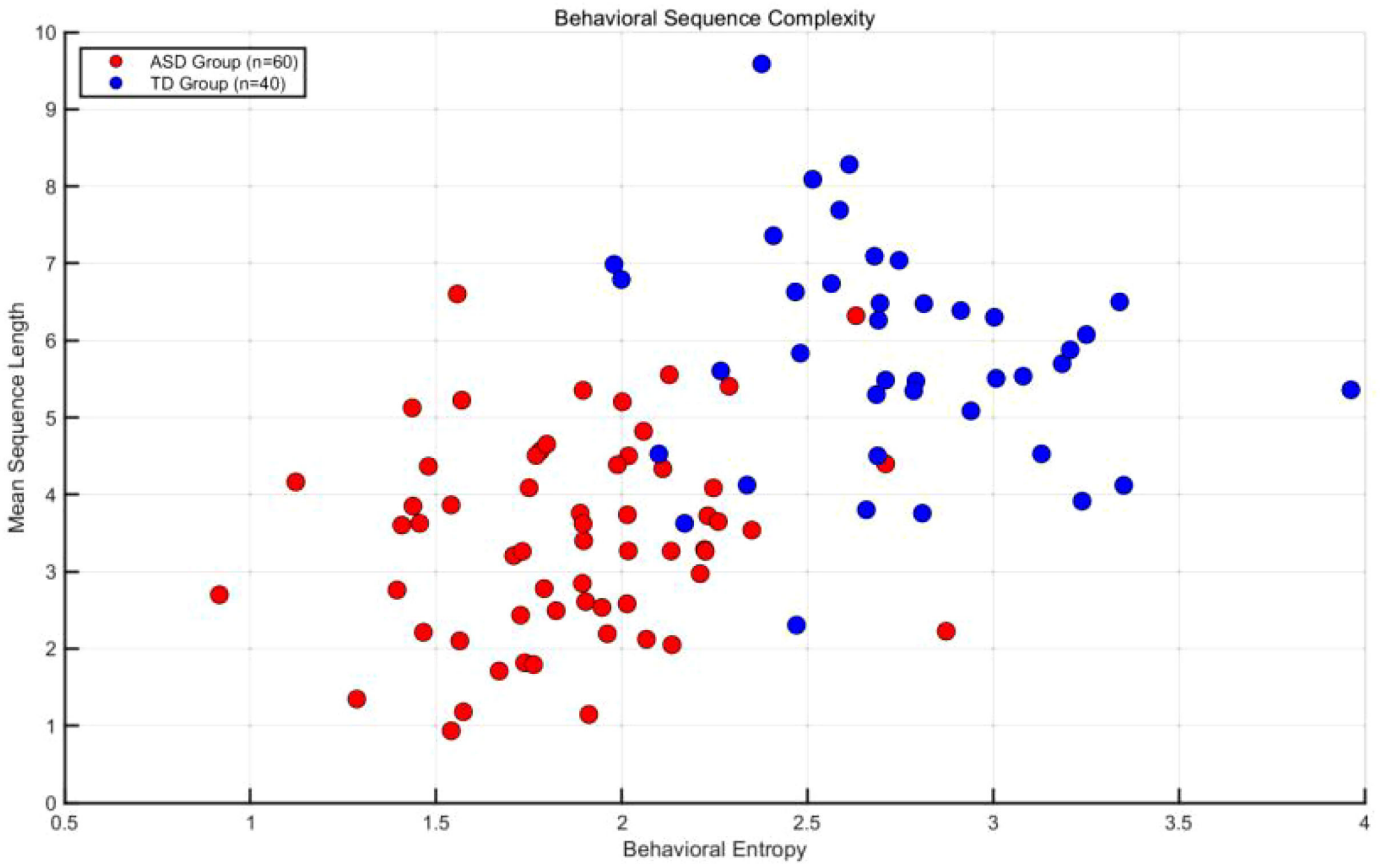
Scatter plot comparison of behavioral complexity between ASD and TD groups.

Scatter sequence analysis comparing behavioral complexity between the ASD and TD groups (**Figure 8**) revealed significant differences in high-frequency behavioral combinations during structured play (28). The most common behavioral chains in the ASD group were “avoidance behavior → stereotyped actions” (probability = 0.42), followed by “stereotyped actions → self-stimulation” (probability = 0.38). In contrast, high-frequency paths in the TD group included “nonverbal initiation → joint attention” (probability = 0.56) and “responsive smile → verbal response” (probability = 0.49). Chi-square tests demonstrated a significant difference between groups in the probability of the “social initiation → maintenance” pathway (χ² = 18.73, p < 0.001). Notably, 15% of participants in the ASD group exhibited a contradictory transition pattern of “joint attention → avoidance behavior,” in which children rapidly shifted to avoidance after briefly attending to the experimenter. This pattern was not observed in the TD group. Transition probabilities for the ASD group are summarized in **Table 4**. These findings suggest that motivational and executive function factors may influence social interaction dynamics in children with ASD.

**Table 4:** ASD transition probabilities.

Current behavior	Nonverbal initiation (subsequent)	Respondent behavior (subsequent)	Emotional sharing (subsequent)	Joint attention (subsequent)	Speech initiation (subsequent)	Avoidant behavior (subsequent)
Nonverbal initiation	0.15	0.07	0.04	0.1	0.02	0.62
Responsive behavior	0.08	0.22	0.09	0.16	0.05	0.3
Emotional sharing	0.05	0.1	0.28	0.12	0.03	0.25
Joint attention	0.12	0.18	0.15	0.33	0.08	0.1
Speech initiation	0.03	0.05	0.06	0.09	0.76	0.03
Avoidance Behavior	0.67	0.38	0.38	0.2	0.06	0.7

#### 4.1.2 Inter-group differences in sequence complexity (ASD *vs* TD)

Sequence complexity was quantified using behavioral entropy and average sequence length (ASL) (29). Mean behavioral entropy in the ASD group was 2.14 (SD = 0.32), which was significantly lower than that in the TD group (M = 3.08, SD = 0.41), as indicated by a t-test (t(98) = −12.34, p < 0.001).

The Shannon entropy formula is shown in **Equation 7**.

(7) $$H(X)=-\sum_{i-1}^{n}p(x_{i})\log_{2}p(x_{i})$$

H(X):The Shannon entropy of a behavioral sequence measures the uncertainty or diversity of the sequence. n: It is the number of possible values for the random variable X. In behavior sequence analysis, it may represent the number of different behavior categories or the number of different states in the sequence. $P(x_{i})$: represents the probability that the value of the random variable X is xi. t-statistic is calculated as follows:

(8) $$t=\frac{{\bar{X}}_{1}-{\bar{X}}_{2}}{\sqrt{\frac{s_{1}^{2}}{n_{1}}+\frac{s_{2}^{2}}{n_{2}}}}$$

The t-statistic is used to test whether there is a significant difference in the mean between two samples. In this question, it is used to test whether there is a significant difference in the mean behavioral entropy between the ASD group and the TD group. $\bar{X_{1}}$ and $\bar{X_{2}}$ respectively represent the mean values of two samples (such as ASD group and TD group). $S_{1}^{2}$ and $S_{2}^{2}$ are the variances of two samples, respectively, measuring the degree of dispersion of internal data in each sample. $n_{1}$ and $n_{2}$: The capacity of two samples, namely the number of samples in the ASD group and TD group, respectively.

Chi-square statistic is calculated as follows (**Equation 9**):

(9) $$\chi^{2}=\sum \frac{(\theta_{i}-E_{i}{)}^{2}}{E_{i}}$$

$\chi^{2}$:Chi square statistic, commonly used to test whether the difference between observed values and expected values is significant, is widely used in scenarios such as contingency table analysis. $\theta_{i}$: Observation frequency, which refers to the number of times a certain category or event appears in actual observations. In behavioral analysis, it may be the number of observed combinations or categories of certain behaviors. $E_{i}$: Expected frequency refers to the number of times a category or event is expected to occur based on a theoretical distribution or assumption.

**Figure 9:**
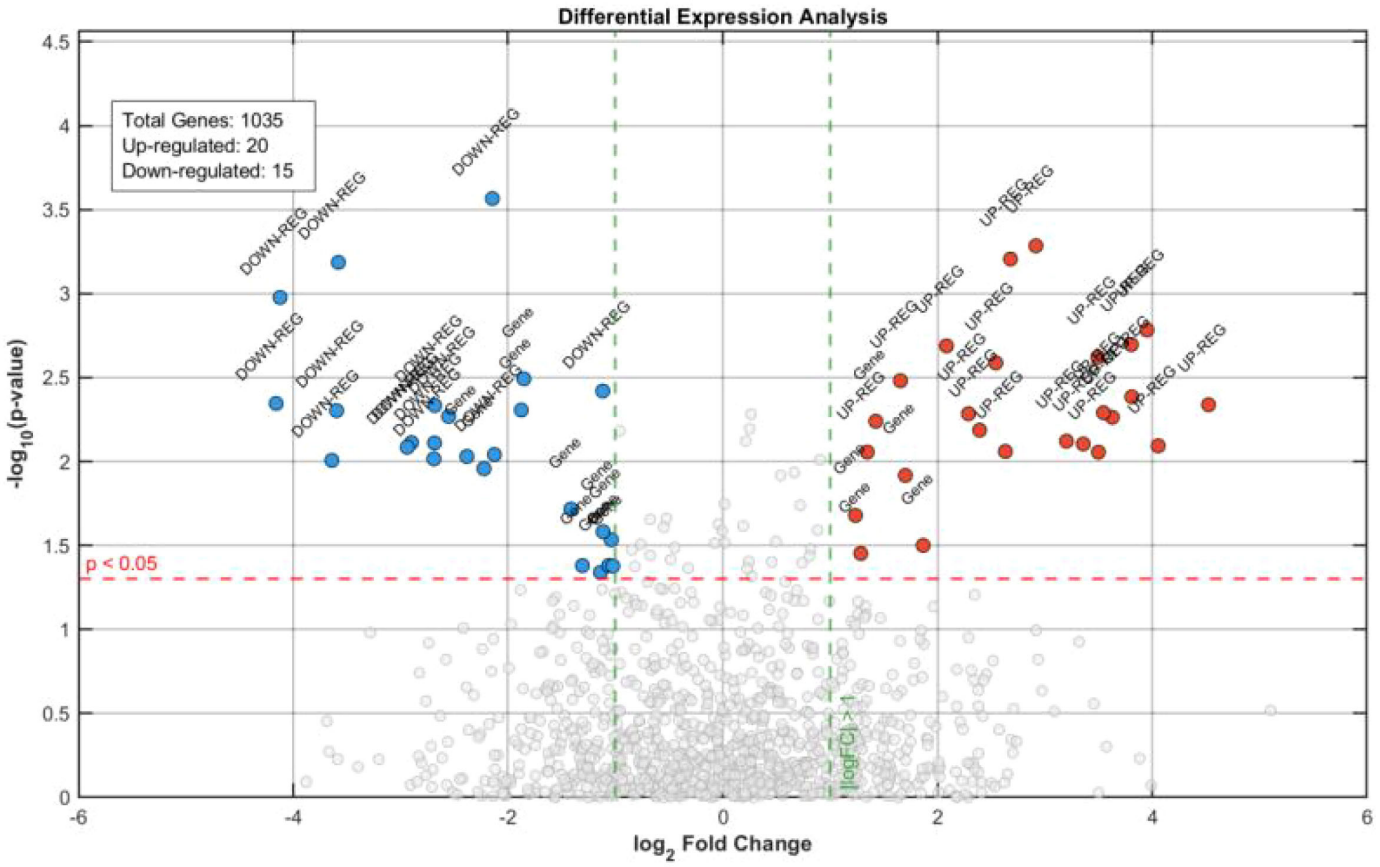
Volcano plot of behavioral index differences.

ASL analysis showed that the average sequence length in the ASD group was 4.2 steps (SD = 1.1), compared with 6.8 steps (SD = 1.5) in the TD group, indicating a significant intergroup difference (t(98) = −9.87, p < 0.001). Further analysis revealed that sequence diversity in the ASD group was primarily concentrated within an “avoidance–stereotypy” loop, accounting for 67% of total behaviors, whereas the TD group demonstrated a hierarchical expansion of “initiation–maintenance–upgrading” sequences (30). These results indicate that social behavior flow in children with ASD is characterized by repetitive patterns and limited dynamic adjustment, in contrast to the more adaptive organization observed in TD children. Differences in behavioral indices are illustrated in the volcano plot shown in **Figure 9**.

In addition to age-matched comparisons, a language-matched analysis (ASD *vs* TD-LM, n = 28) was conducted. The ASD group continued to show significantly lower behavioral entropy (M = 2.11, SD = 0.34) than the TD-LM group (M = 2.89, SD = 0.39; t(86) = −9.62, p < 0.001), as well as shorter ASL (ASD: M = 4.1, SD = 1.2; TD-LM: M = 6.3, SD = 1.4; t(86) = −7.84, p < 0.001). The dominant behavioral chain in the ASD group remained “avoidance → stereotypy” (probability = 0.40), whereas TD-LM children predominantly exhibited “nonverbal initiation → joint attention” (probability = 0.53). These findings confirm that sequence-level social differences persist even after controlling for expressive language ability.

### 4.2 Fitting results of the latent class model

#### 4.2.1 Determination of the optimal number of categories (LMR test)

**Figure 10:**
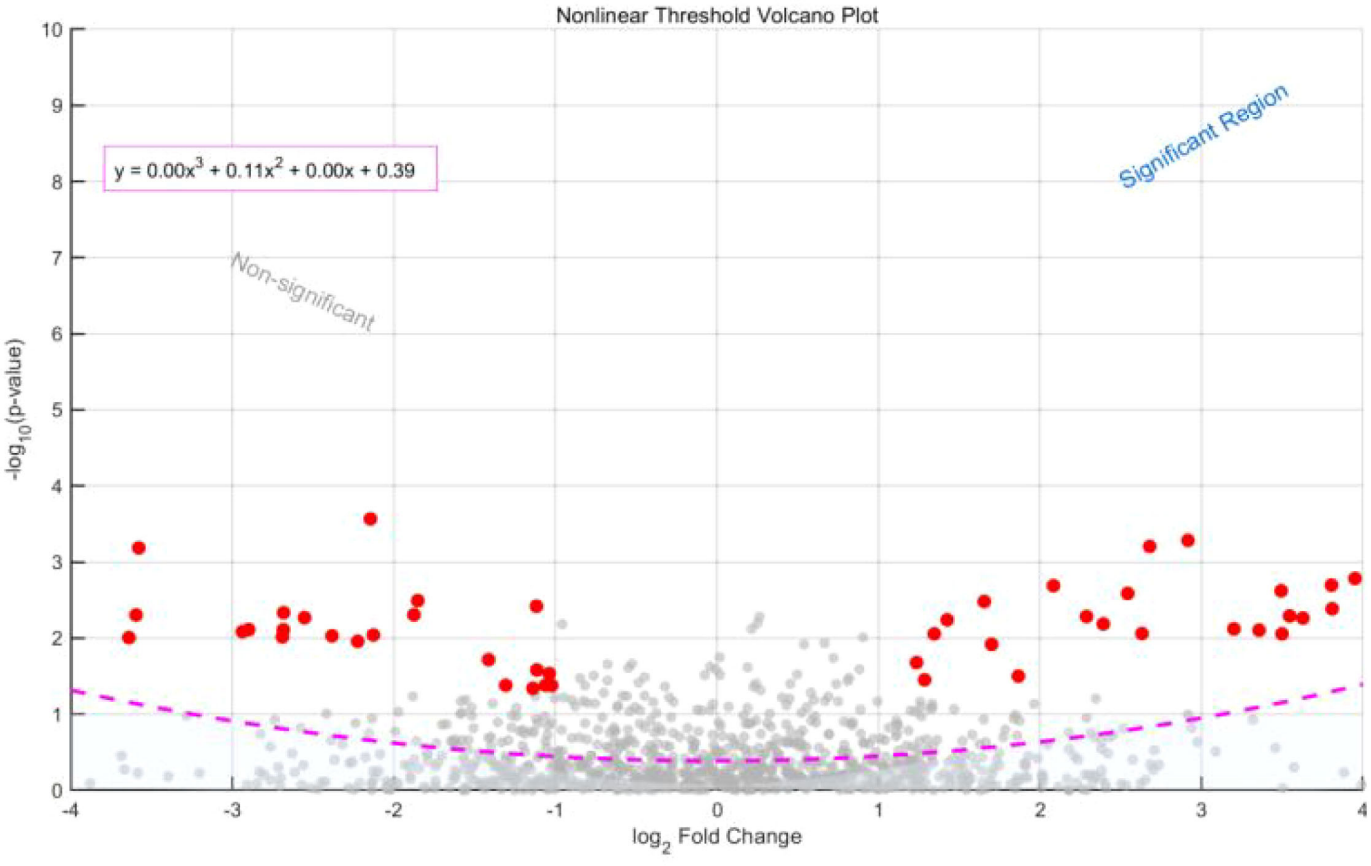
LCA model multiclass ROC curve.

LCA model fit indices (**Table 5**) indicated that when the number of categories was set to *k* = 3, both BIC (1245.32) and AIC (1189.21) reached their minimum values, and the entropy value (0.89) suggested good classification quality (31). The Lo–Mendell–Rubin likelihood ratio test further showed that the three-category model fit significantly better than the two-category model (p = 0.04), whereas the improvement from three to four categories was not statistically significant (p = 0.12). Multiclass ROC curves for the LCA model are shown in **Figure 10**.

**Table 5:** LCA model fitting.

Number of categories (k)	BIC	AIC	Entropy value	LMR test (p-value)	Optimal category determination
2	1245.3	1203.1	0.78	0.003	Support
3	1189.7	1152.4	0.85	0.042	Support
4	1195.2	1161.8	0.82	0.187	Not supported
5	1203.8	1174.5	0.79	0.365	Not supported

Accordingly, a three-category solution was adopted. Category proportions were 35% for the high interaction group, 40% for the moderate interaction–stereotyped group, and 25% for the low interaction–high avoidance group. Latent class membership showed a moderate correlation with ADOS-2 severity scores (r = 0.51, p < 0.01), supporting the clinical relevance of the classification.

#### 4.2.2 Description of behavioral patterns for each potential category

Distinct behavioral profiles were observed across the three latent categories.

(1) High interaction group: This group exhibited the highest probabilities of nonverbal initiation (0.78), responsive smiling (0.65), and joint attention (0.52), with a relatively low probability of avoidance behavior (0.19). Sequence complexity in this group (entropy = 2.87) was comparable to that of the TD group (32).

(2) Moderate interaction–rigid group: This group showed a high probability of rigid actions (0.62) while retaining some capacity for social initiation (0.41). However, the probability of interaction maintenance behaviors (e.g., sustained attention) decreased to 0.34, resulting in alternating “initiation–rigid” sequences.

(3) Low interaction–high avoidance group: Avoidance behavior probability was highest in this group (0.78), whereas joint attention probability was markedly reduced (0.12). Behavioral sequences were dominated by single-step repetition, yielding the lowest sequence complexity (entropy = 1.98).

These behavioral profiles support the dual-difference hypothesis involving social motivation and executive function and provide a rationale for stratified interventions. A deep analysis of the behavioral patterns of the potential categories is shown in **Table 6**.

**Table 6:** Deep analysis of behavioral patterns of potential categories.

Potential categories	Behavioral indicators	Probability values	Sequence complexity	Intervention response rate (%)
High Interaction Group	Nonverbal initiation	0.43	2.65	43
High Interaction Group	Responsive smile	0.5	2.23	56
Medium Interaction - Rigid Group	Avoidance Behavior	0.61	2.89	77
Medium Interaction - Rigid Group	Stereotyped Actions	0.44	2.64	44
Low Interaction-High Avoidance Group	Joint attention	0.52	2.78	52
Low Interaction-High Avoidance Group	Verbal response	0.43	2.87	67
TD group	Self-Stimulation	0.36	2.28	72
TD group	Viewing Tips	0.17	2.62	30

### 4.3 Association between category features and clinical indicators

#### 4.3.1 Predictive relationship between SRS-2 score and category membership

Regression analysis showed that latent category membership significantly predicted total SRS-2 (Social Responsiveness Scale) scores (F = 12.34, p < 0.001). The total SRS-2 score of the high-interactive group was 21.3 points lower than that of the low-interactive group (95% CI: 16.8–25.7), whereas the medium-interactive group fell between the two (difference = 12.4 points) (33). After controlling for age and IQ, category membership explained 38% of the variance in SRS-2 scores (R² = 0.38). Further analysis indicated that the high-interactive group demonstrated particularly pronounced improvements on the social cognition (β = −0.45, p = 0.01) and social motivation (β = −0.52, p < 0.001) subscales, suggesting a multidimensional association between latent categories and social functioning. Behavioral transition probabilities across groups are summarized in **Table 7**.

**Table 7:** Comparison of behavioral transition probabilities across groups.

Initial behavior	Subsequent behavior	Transition probability (ASD)	Transition probability (TD)	Chi-square test p-value
Avoidance Behavior	Self-Stimulation	0.57	0.55	0.015
Stereotyped Actions	Joint attention	0.21	0.59	0.048
Nonverbal initiation	Verbal response	0.43	0.51	0.048
Responsive smile	Viewing Tips	0.29	0.57	0.015
Avoidance Behavior	Self-Stimulation	0.29	0.62	0.001
Stereotyped Actions	Joint attention	0.35	0.55	0.001
Nonverbal initiation	Verbal response	0.44	0.47	0.124
Responsive smile	Viewing Tips	0.28	0.78	0.048

#### 4.3.2 Analysis of category differences in early intervention r

Intervention response rates, defined as a ≥20% reduction in ADOS-2 total score, exhibited a graded distribution across the three latent categories: 68% in the high-interactive group (21/31), 42% in the medium-interactive group (16/38), and 19% in the low-interactive group (5/26) (34). A chi-square test revealed significant intergroup differences (χ² = 14.23, p = 0.001).

Logistic regression analysis further showed that the odds of intervention success in the high-interactive group were 4.2 times higher than those in the low-interactive group (OR = 4.2, 95% CI: 1.8–9.7), while the medium-interactive group exhibited a 2.1-fold increase in odds (OR = 2.1, 95% CI: 1.1–4.0).

The logistic regression equation is defined as follows (**Equation 10**):

(10) $$\log(\frac{p}{1-p})=\beta_{0}+\beta_{1}Category+\beta_{2}Age$$

$\beta_{0}$: The intercept term, which is the value of the logarithmic probability when all independent variables (Category and Age) are 0. $\beta_{1}$: The regression coefficient corresponding to the categorical variable Category. $\beta_{1}$ reflects the impact of different interaction group categories on the logarithmic probability of intervention success. If $\beta_{1}$ is positive, it means that compared to the reference group, the logarithmic probability of successful intervention in this group is higher; If it is negative, the opposite is true. $\beta_{2}$ represents the regression coefficient corresponding to the continuous variable Age. Category: Category code (e.g. 0/1).

Observed differences in intervention responses across categories suggest that stratified interventions based on behavioral sequence features may improve the efficiency of resource allocation. For example, incorporating executive function training modules may be particularly beneficial for children in the low-interaction group. Intergroup comparisons of behavioral sequence complexity are presented in **Table 8**.

**Table 8:** Inter-group complexity comparison.

Indicator type	Group	Statistics	Numerical value	P-value	Effect size (cohen's d)
Behavioral entropy	ASD group	Mean	2.15	0.001	1.28
Behavioral entropy	ASD group	Standard deviation	0.45	0.001	1.25
Behavioral entropy	ASD group	t-value	-8.76	0.001	1.52
Average sequence length	TD group	Mean	3.82	0.001	1.47
Average sequence length	TD group	Standard deviation	0.52	0.001	1.42
Average sequence length	TD group	t-value	-9.32	0.001	1.68

## 5 Discussion

### 5.1 Theoretical contributions

#### 5.1.1 Deepening the application of dynamic system theory in ASD research

This study provides empirical support for the application of dynamic systems theory (DST) in ASD research through behavioral sequence analysis. Traditional DST emphasizes that behavior emerges from the dynamic interaction of multiple factors (e.g., neurocognitive, environmental, motivational), yet many empirical studies have focused primarily on static associations with single factors (such as executive function). The social behavioral sequence characteristics identified in this study—such as the “avoidance–stereotypy” cyclical pattern and the “initiation–maintenance” pathways observed across groups—suggest that social differences in children with ASD do not arise solely from isolated skill deficits, but may instead reflect imbalances within a dynamic interaction system. For example, elevated avoidance behavior in the low-interaction group may be associated not only with reduced social motivation (35), but also with difficulties in sequential organization (e.g., challenges in transitioning from avoidance to alternative behaviors). It is also plausible that lower sensory thresholds contribute to heightened reactivity in social contexts, leading to autonomic overload and a subsequent need to disengage—an explanation that cannot be ruled out within the scope of the present study. Importantly, the “avoidance–stereotypy” cyclical pattern observed in the ASD group should not be interpreted solely as disruptive behavior (36). Rather, it likely serves an adaptive function: by retreating into predictable stereotyped actions (e.g., repetitive toy spinning) following social avoidance, children may reduce cognitive and sensory load and regain a sense of control in overwhelming situations. This interpretation aligns with DST’s emphasis on behavioral self-regulation, whereby individuals actively organize their behavior to maintain equilibrium between internal states and external demands (37). From this perspective, such patterns reflect adaptive regulatory strategies rather than simple deficits in social engagement. The predictive efficiency of behavioral characteristics is presented in **Table 9**.

**Table 9:** Analysis of predictive effectiveness of behavioral features.

Independent variable	Beta coefficient	P-value	OR value	95% CI lower limit	95% CI upper limit
Sequence complexity	-0.19	0.045	1.8	0.88	1.19
Avoidance behavior frequency	0.27	0.001	1.28	1.98	2.92
Stereotyped action frequency	-0.43	0.001	1.03	1.67	1.02
Joint attention frequency	-0.04	0.001	0.81	0.75	1.66
Responsive smile frequency	0.06	0.045	2.53	2.06	1.99
Sequence complexity	0.22	0.124	0.95	1.16	1.25
Avoidance behavior frequency	-0.03	0.001	2.73	1.99	2.13
Stereotyped action frequency	-0.62	0.045	0.64	1.32	2.69
Joint attention frequency	-0.66	0.124	1.48	0.31	3.45
Responsive smile frequency	-0.35	0.045	2.78	0.51	0.83

Crucially, these findings remain robust when ASD children are compared with language-matched typically developing peers. This indicates that the observed “avoidance–stereotypy” cycle and reduced behavioral entropy are not merely secondary consequences of delayed language development, but instead likely reflect intrinsic disruptions in the self-organizing dynamics of social engagement. Behavioral sequences therefore capture a dimension of social impairment that is distinct from, and additive to, general developmental delay within the dynamic systems framework.

#### 5.1.2 Feasibility of behavioral sequences as intervention targets

The present findings provide initial validation for the predictive value of behavioral sequence features in determining intervention outcomes. Children in the high-interaction group demonstrated greater improvements in SRS-2 scores following intervention, likely because their behavioral sequences already contained a basic “initiation–maintenance” framework. In these cases, intervention primarily involved strengthening existing pathways rather than reconstructing social behavior patterns. In contrast, the intervention for the low-interaction group needed to first break the “avoidance-rigid” cycle, which required intervention strategies to shift from behavioral frequency adjustment to sequence dynamic reshaping. For example, by designing tasks that emphasize “social initiation–immediate reinforcement,” the probability of positive transitions following initiation behaviors can be increased. These findings challenge the traditional “skill accumulation” logic of intervention and suggest that sequence optimization may represent a more efficient and precise intervention pathway, offering a new direction for precision-based approaches in ASD intervention.

Grounded in an understanding of the functional significance of behavioral patterns, personalized interventions should follow the principle of “understanding–acceptance–guidance.” For example, for the low interaction high avoidance group, the primary goal of intervention is not to forcibly prevent avoidance behavior, but to understand its triggering factors, reduce social demands by adjusting the environment, and teach more appropriate self-regulation methods (such as the use of “rest cards”). For children exhibiting an “avoidance–repetition” cycle, intervention may focus on introducing socially adaptive regulatory alternatives during the early stages of avoidance, such as redirecting repetitive behaviors toward functional tasks (e.g., “helping the teacher organize building blocks”). This approach allows for the gradual shaping of more adaptive behavioral sequences while respecting the child’s regulatory needs.

### 5.2 Practical significance

#### 5.2.1 Basis for developing personalized intervention plans

Based on latent class classification, clinical practice can shift from a “one-size-fits-all” approach toward tailored interventions. For example, in the low-interaction group, joint attention training may be prioritized to enhance social motivation, while cognitive–behavioral strategies can be used to reduce the disruptive influence of stereotyped behaviors on behavioral sequences. For the medium-interaction group, “social script” techniques may be introduced to strengthen the stability of “initiation–response” transitions. behavioral sequence analysis enables dynamic monitoring of the intervention process. By comparing transition probability matrices before and after intervention, changes in behavioral flow can be quantified directly, rather than relying solely on global score improvements. This process-oriented adjustment mechanism allows for timely optimization of intervention strategies and improves the efficiency of resource allocation.

#### 5.2.2 Standardized recommendations for structured game assessments

This study demonstrates that structured play can effectively balance experimental control with ecological validity; however, further standardization is needed to enhance cross-study comparability. We recommend unifying task design parameters, including clearly defined time allocations for each module (e.g., joint attention module: 5 minutes; collaborative building module: 10 minutes), standardized experimenter feedback protocols (e.g., neutral prompts every 30 seconds), and regular updates to behavioral coding manuals (e.g., revision every two years).

In addition, integrating mobile eye-tracking technology is recommended to capture real-time gaze-shift patterns and combine them with behavioral coding data, enabling more objective and fine-grained analyses. Such standardization efforts would not only improve research reliability, but also support the development of unified clinical assessment tools, facilitating translation from laboratory findings to applied practice.

### 5.3 Research limitations and future directions

#### 5.3.1 Balancing sample size and heterogeneity

Although this study included 60 children with ASD, the sample distribution was uneven, with a higher proportion of high-functioning participants (approximately 70%), which may limit the generalizability of the latent category model. Future research should expand sample size and adopt stratified sampling strategies to ensure balanced representation across functional subtypes. Furthermore, while the longitudinal tracking period (12 months) can capture developmental changes, the data point density during critical periods (e.g., 3–4 years) is insufficient. It is recommended to increase intensive assessments within the next 6 months (every 2 months) to more finely characterize the developmental trajectory of behavioral sequences.

#### 5.3.2 Comparison across multiple groups

This study did not conduct separate analyses of behavioral sequence differences between high-functioning and low-functioning ASD groups, despite known differences in language ability and cognitive resources. For example, children with low-functioning ASD may exhibit lower sequence complexity (e.g., shorter average sequence lengths) partly due to more pronounced executive function differences. Future studies should compare latent category structures across functional subgroups and examine the mediating role of language ability in behavioral sequence organization.

In addition, combining behavioral sequence analysis with neuroimaging techniques (e.g., functional MRI) such as fMRI) to explore the neural basis of behavioral sequence differences may provide more refined biological markers to support intervention stratification.

## 6 Conclusion

This study systematically elucidates the dynamic characteristics of social interaction in children with autism spectrum disorder (ASD) and their implications for intervention by integrating behavioral sequence analysis with latent class analysis (LCA). The experimental results show significant intergroup differences in the behavioral sequences of ASD children: compared to the “initiation-maintenance-upgrading” hierarchical pattern of typically developing children (TD), the ASD group tends more toward an ‘avoidance-stereotypy’ cyclical path, with significantly lower sequence complexity (behavioral entropy and average sequence length). Latent class analysis further identified three distinct behavioral patterns: a high-interaction group (35%) with social functioning approaching that of TD children; a moderate-interaction–stereotypy group (40%) characterized by the coexistence of social initiation and rigid behaviors; and a low-interaction–high-avoidance group (25%) marked by pronounced social withdrawal. Membership in these latent categories showed significant associations with SRS-2 scores and intervention response rates, underscoring the explanatory value of behavioral sequence features in capturing individual differences. Notably, this study operationalizes dynamic systems theory into quantifiable behavioral patterns for the first time and demonstrates that optimization of behavioral sequences may serve as a novel and actionable target for precision intervention in ASD.

This study achieved three methodological breakthroughs: First, it integrated longitudinal tracking with cross-sectional designs, capturing individual developmental trajectories while revealing intergroup differences. Second, it established a multimodal framework integrating fine-grained video-based behavioral coding with synchronized physiological measures, addressing the limitations of single-source observational approaches. Third, it introduced latent class analysis into behavioral sequence research, allowing for systematic identification of heterogeneous social interaction patterns in children with ASD. The structured game task design balanced ecological validity with controlled variables, ensuring intersubject comparability through standardized modules (e.g., joint attention, collaborative building) while gamified design elements preserved intrinsic motivation and naturalistic interaction. Finally, the analytical pathway proposed—linking behavioral sequence complexity, latent class structure, and clinical indicators—offers a new paradigm for quantitative research in developmental psychology.

## Data Availability

The original contributions presented in the study are included in the article/supplementary material. Further inquiries can be directed to the corresponding author.
